# Supplementary material for: The Gastroprotective Effects of Anisomeles indica against Ethanol-Induced Gastric Ulcer through the Induction of IκB-α and the Inhibition of NF-κB Expression
Source: Nutrients. 2024 Jul 17;16(14):2297. doi: 10.3390/nu16142297 (PMC11279643; doi:10.3390/nu16142297)
Supplement: Supplementary file 1 [file nutrients-16-02297-s001.zip › nutrients-3084406-supplementary.pdf]

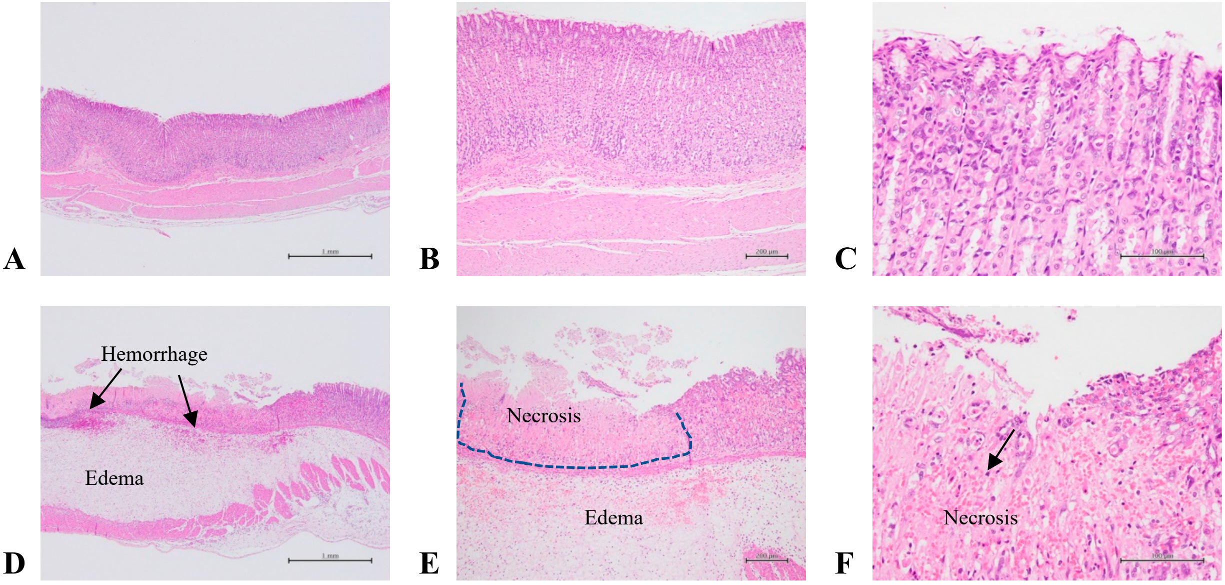

Figure S1A. Histopathological changes of the stomach in the protective effects of *A. indica* HP813 powder on gastric mucosa in rats - control and positive control group. No significant change of the stomach was noted in the control (animal code: 107). However, 70% ethanol caused multifocal slight to moderate/severe degeneration/ necrosis with hemorrhage, inflammation and edema and hemorrhage in the mucosa of the stomach in the positive control group (animal code: 205). The severity score of necrosis was 0 and 2.9±0.9 in the control and positive control groups, respectively. H&E stain, 40x, 100x, 400x. Arrow.

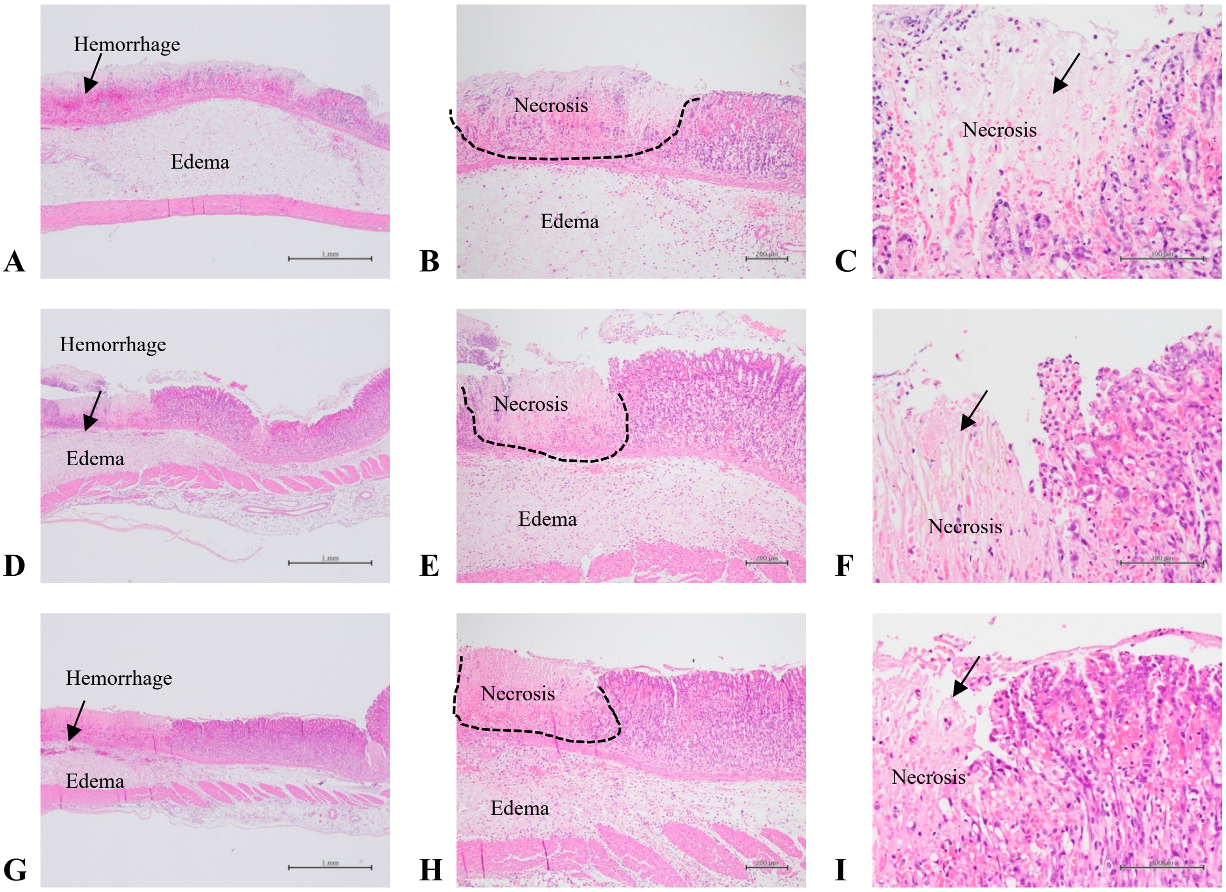

Figure S1B. Histopathological changes of the stomach in the protective effects of *A. indica* HP813 powder on gastric mucosa in rats – low, middle and high-dose group. The 70% ethanol caused multifocal minimal to moderate/severe degeneration/necrosis with hemorrhage, inflammation and edema and hemorrhage in the mucosa of the stomach in the low (A-C. animal code: 302), middle (D-F. animal code: 409) and high-dose (G-I. animal code: 508). The severity score of necrosis was 2.9±0.8, 2.4±0.7 and 2.3±0.8 in the low, middle and high-dose groups, respectively. H&E stain, 40x, 100x, 400x. Arrow.

Table S1. The IHC intensity of immune reaction by semi-quantitatively analyze.

	Intensity		
	TNF-α	NF-κB	IκB-α
Control	1.14 ± 0.03	2.27 ± 0.01	3.26 ± 0.06
Positive control	2.30 ± 0.03	3.29 ± 0.04	2.17 ± 0.02
HP813 Low dose	2.00 ± 0.09*	3.02 ± 0.07*	2.78 ± 0.04*
HP813 Medium dose	1.74 ± 0.06*	2.64 ± 0.07*	3.07 ± 0.19*
HP813 High dose	1.35 ± 0.07*	2.48 ± 0.02*	3.27 ± 0.05*

* Statistically significant difference between the positive control and treatment groups at $p < 0.05$.
